# Two cases of cardiac device-related endocarditis due to Streptococcus dysgalactiae subsp. equisimilis (group C or G streptococci)

**DOI:** 10.1186/1471-2334-14-174

**Published:** 2014-03-29

**Authors:** Sari Rantala, Suvi Tuohinen

**Affiliations:** 1 Department of Internal Medicine, Tampere University Hospital, P.O. Box 2000, FIN-33521 Tampere, Finland; 2 Heart Center Co., Tampere University Hospital and School of Medicine, University of Tampere, Tampere, Finland

**Keywords:** *Streptococcus dysgalactiae* subsp. *equisimilis*, Group G streptococci, Cardiac device, Endocarditis, Lead extraction

## Abstract

**Background:**

Cardiac device-related endocarditis is a very rare clinical manifestation of *S. dysgalactiae* subsp. *equisimilis* disease. This pathogen is a common cause of cellulitis. We here report two cases of cardiac device-related endocarditis due to *Streptococcus dysgalactiae* subsp. *equisimilis*. Blood cultures yielded this pathogen and both patients had recurrent bacteremia. Transthoracic and transesophageal echocardiography revealed lead vegetations. This is a new description of this pathogen to cause cardiac device-related endocarditis.

**Case presentation:**

The first case is a 79-year-old finnish woman who received a dual-chamber pacemaker for intermittent complete heart block in April 2011. She had three episodes of *S. dysgalactiae* subsp. *equisimilis* bacteremia. During first episode she had arthritis of glenohumeral joint. Focus was unknown in the second and third bacteremic episodes. During third bacteremic episode transesophageal echocardiography (TEE) revealed lead vegetation. Patient underwent successful complete system removal. She was treated with benzylpenicillin four million IU six times a day for four weeks intravenously. The second case is a 92-year-old finnish man. A dual-chamber pacemaker was implanted on June 2012 due to total heart block. He had recurrent *S. dysgalactiae* subsp. *equisimilis* bacteremia with cellulitis. During the second bacteremic episode transthoracic echocardiography (TTE) was performed because of persistent fever. Echocardiography revealed lead vegetation. Abdominal CT revealed also an abscess in the psoas region. This elderly patient was very fragile, and the pacemaker system was not extracted. Therapy was continued with benzylpenicillin four million IU six times a day for six weeks intravenously and thereafter suppressive treatment with amoksisillin 500 mg three times a day was initiated.

**Conclusion:**

*Streptococcus dysgalactiae* subsp. *equisimilis* (group C and G streptococci) seldom cause cardiac device endocarditis. Both patients had recurrent bacteremia of *S. dysgalactiae* subsp. *equisimilis* and echocardiography revealed cardiac device-related endocarditis*.* These cases emphasize the importance of considering endocarditis in elderly persons having cardiac devices together with the presence of unexplained bacteremia, fever without focus or persistent fever.

## Background

According the current taxonomy, the beta-hemolytic streptococci are divided into Lancefield group A streptococcus (*S. pyogenes*), Lancefield group B streptococcus (*S. agalactiae*) and Lancefield group C and G streptococci (*S. dysgalactiae* subsp. *equisimilis*). The spectrum of *S. dysgalactiae* subsp. *equisimilis* diseases ranges from pharyngitis, tonsillitis and skin and soft-tissue infections such as wound infections, erysipelas and cellulitis to life-threatening necrotizing fasciitis and streptococcal toxic shock syndrome [1]. It can also cause pneumonia, septic arthritis, osteomyelitis, meningitis, endocarditis, puerperal sepsis and bacteremia without focus [1]. Pharyngitis and tonsillitis are the most frequent non-invasive manifestations of *S. dysgalactiae* subsp. *equisimilis* disease. Cellulitis is the most common clinical manifestation in *S. dysgalactiae* subsp. *equisimilis* bacteremia [1]. This pathogen constitutes a major cause of illness in elderly patients with underlying diseases and skin breakdown [1,2].

During the past twenty years the use of implantable cardiac devices has increased in the United States [3]. TEE and blood culture are the most important diagnostic tools in cardiac device-related endocarditis. This condition remains a rare but potentially lethal complication of device implantation [4]. Here we describe two cases of cardiac device-related endocarditis due to *S. dysgalactiae* subsp. *equisimilis* hospitalized in Tampere University Hospital. Both patients had recurrent bacteremia of *S. dysgalactiae* subsp. *equisimilis* origin and echocardiography revealed cardiac device-related endocarditis. These two cases emphasize the importance of considering endocarditis in adults having cardiac devices together with the presence of unexplained bacteremia or fever without focus.

## Case presentation

### Case 1

A 79-year-old woman received a dual-chamber pacemaker for intermittent complete heart block in April 2011. She was on nifedipin 30 mg daily dose for hypertension. She was admitted to the district hospital in Vammala on January 2013 and diagnosed with *S. dysgalactiae* subsp. *equisimilis* septicemia. The focus of bacteremia was unknown. On examination she was feverish and presented rubor and swelling in her tender glenohumeral joint. The arthritis was considered to be reactive, and no aspiration of joint fluid was made. She completed a 13-day course of cefuroxime.

In February 2013 she developed recurrent *S. dysgalactiae* subsp. *equisimilis* septicemia and was treated in the district hospital in Vammala. The focus of bacteremia was still unknown. The only symptom was the articular pain in the right glenohumeralis joint, this time without redness or swelling. The electrocardiogram showed sinus rhythm, and there were normal auscultatory findings. Full-body CT showed pericardial and pleural effusion. TTE revealed no vegetation. She received a treatment course of ceftriaxone intravenously for 10 days.

**Figure 1:**
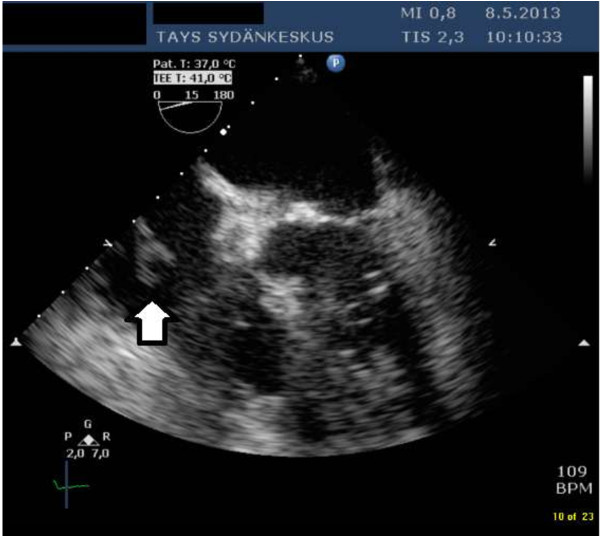
**Transesophageal echocardiography examination of the first patient.** The bold arrow points to the two-tailed vegetation in the right atrium. The vegetation was attached to the pacemaker lead.

On the 5th of May 2013 she was admitted to Tampere University Hospital with fever and paresis of the right radialis nerve. There was a new systolic murmur heard on the apex. CT of the brain was normal. Two cultures of blood taken on admission yielded *S. dysgalactiae* subsp. *equisimilis.* Laboratory test results revealed normal hemoglobin level of 129 g/liter, an elevated leucocyte count of 14× 10^9^ /liter, and an elevated C-reactive protein level of 140 mg/liter. TEE revealed a 15 mm long mass attached to the pacemaker lead on the right atrium (Figure 1). There was a good cardiac function with ejection fraction over 60 per cent, and no signs of valvular vegetations. She was treated with benzylpenicillin at four million IU six times a day. On 16th of May the patient underwent successful complete pacemaker removal. A temporary pacemaker system was implanted due to intermittent total heart block. Samples from the leads extracted yielded no growth. She completed a four-week course of intravenous antibiotics and has subsequently remained well. A new permanent pacemaker was implanted uneventfully on the right side in June 2013.

### Case 2

On 30th of July 2013 a 92-year-old man was admitted to Hatanpää City Hospital with fever and cellulitis of the arm. He had a prior history of hypertension, paroxysmal atrial fibrillation, polymyalgia rheumatica and a prosthetic knee joint on right side inserted in the year 2008. A dual-chamber pacemaker was implanted in June 2012 due to total heart block. His regular treatment consisted of prednisolon 2.5 mg, furosemid 20 mg, nilvadipin 8 mg, aspirin 100 mg, pantopratsol 40 mg, calcium and vitamin D supplement, magnesium supplement 250 mg, and gabapentin 900 mg daily doses. Blood culture revealed *S. dysgalactiae* subsp. *equisimilis* bacteremia and he suffered from cellulitis of the arm. He was treated at Hatanpää City Hospital.

On 30th of September 2013 he was again admitted to Hatanpää City Hospital. On admission his temperature was 38.3°C. The serum blood test revealed reduced hemoglobin level of 91 g/liter, an increased leucocyte count of 20 × 10 10^9^ /l, and an increased C-reactive protein level of 302 mg/liter. Two blood cultures taken on admission were positive for *S. dysgalactiae* subsp. *equisimilis*. He was diagnosed a recurrent septicemia and cellulitis in the right leg. There were no skin lesions. Initial treatment with cefuroxime 1.5 g intravenously three times a day was changed to benzylpenicillin at two million IU six times a day (total 12 million IU a day) after two days.

**Figure 2:**
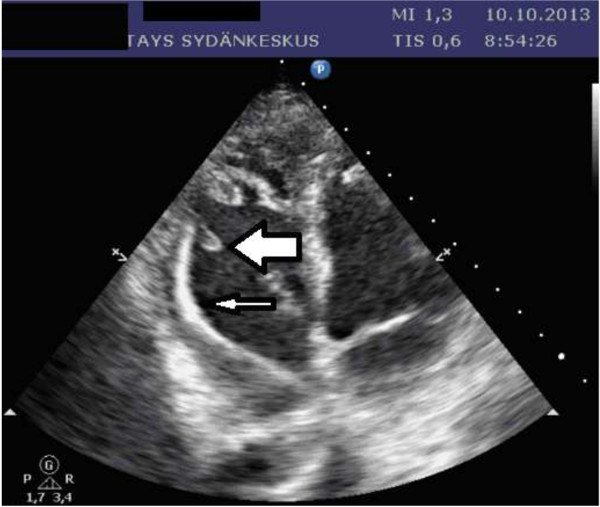
**Transthoracic echocardiography examintion of the second patient.** The slender arrow points to the ventricular pacemaker lead. The bold arrow points to the fluttering vegetation mass.

On 5th of October he was transferred to Tampere University Hospital, because he had persistent fever. He suffered from severe pain in his right foot. He was afebrile but disorientated at arrival, and could not lift his right leg. Blood pressure on admission was 127/64 mmHg. On auscultation there were normal heart sounds. An abdominal CT revealed an abscess in the psoas region on the left side with maximal diameter of 5.8 cm. The aspiration fluid culture was negative for bacteria. A chest X-ray showed pericardium effusion. An echocardiography was performed. The patient appeared quite ill, and was considered not to be eligible for TEE. TTE revealed a large vegetation on the ventricular pacemaker lead. This fluttering mass had diameters of 14 × six mm (Figure 2), and was attached to the pacemaker leads distal end. Pulmonary artery pressure was estimated to 70–75 mmHg, otherwise echocardiograpy results were normal with good biventricular function and no signs of valvular lesions. The patient was treated with benzylpenicillin at four million IU six times a day (total 24 million IU a day) and a 500 mg daily dose of oral levofloxacin.

This elderly patient was very fragile, and the pacemaker system was not extracted. He suffered from back pain and persistent pain in his right buttock. Abdominal CT revealed spondylodiscitis at the level of L4/5. Therapy was continued with benzylpenicillin at four million IU six times a day for six weeks intravenously followed by suppressive treatment with amoksisillin a 500 mg three times a day.

## Conclusion

Cardiac device-related endocarditis is a very rare clinical manifestation of *S. dysgalactiae* subsp. *equisimilis* disease [1,4]. A thorough search of the literature identified no published cases of cardiac device-related endocarditis involving *S. dysgalactiae* subsp. *equisimilis* as causative agents. *Staphylococcus aureus* is the major infective agent responsible for 47% of cardiac device-related endocarditis, followed by coagulase-negative staphylococci (35%), other gram positive (11%) and gram negative (11%) organisms [5]. Endocarditis caused by *S. dysgalactiae* subsp. *equisimilis* is uncommon, accounting for fewer than 1% of total cases [6].

The use of pacemakers has increased in the last 15 years [7,8]. Likewise the number of elderly persons with chronic illness is growing. Both case patients were elderly and *S. dysgalactiae* subsp. *equisimilis* bacteremia usually occurs at high age [9]. In the Pirkanmaa area in Finland the incidence of *S. dysgalactiae* subsp. *equisimilis* bacteremia has increased [10]. These factors might contribute to the occurrence of rare and serious infections caused by this bacterium, such as endocarditis. The second patient was treated with immunosuppressive therapy for his rheumatology disease and that could make him prone to rare pathogen. Recurrent bacteremia is common in *S. dysgalactiae* subsp. *equisimilis* bacteremia [11]. Cellulitis is the most common clinical manifestation in recurrence [11]. *S. dysgalactiae* subsp. *equisimilis* isolates can aggregate human platelets and can internalize into human endothelial cells and they may both promote persistence within the vascular system [12]. Rohde and co-workers have identified a fibronecting-binding protein, GfbA, from group G streptococci, which functions as an adhesin and invasin and the invasion mechanism differ from group A streptococci [13].

A cardiac device -related infection is a life-threatening complication [5,14]. Blood cultures have an important role in the diagnostic process of suspected infective endocarditis. During 2013 the BacT/ALERT 3D (bioMérieux SA, Marcy-L’Etoile, France) blood culture system with standard culture media was used. In the current cases, the Lancefield serogroups were defined by latex agglutination using the Streptex latex test system (Remel Europe Ltd, Dartford, UK). All isolates were also strain identified by a commercial test (Rapid ID 32 STREP, bioMérieux SA, Marcy-1’Etoile, France). Previous studies of permanent pacemaker lead endocarditis have confirmed the superiority of TEE over TTE [4,15]. Management should consist in complete device extraction and long-term administration of antibiotics which cover the pathogen cultured from blood or samples from leads extracted. Cardiac device-related endocarditis is associated with a mortality rate of 66% if the device is not extracted [14]. In the case of complete device extraction combined with antimicrobial therapy the mortality rate is 13-21% [4,14]. The optimal timing of re-implantation remains unknown. Re-implantation should be conducted at a new site. Most authors think that re-implantation can take place when the patients are no longer bacteremic [16]. The duration of antibiotic therapy has varied widely in the published literature [16-18]. Some authors have suggested that four weeks of therapy should be sufficient [16]. Several guidelines recommend the addition of aminoglycoside to penicillin therapy of streptococcal endocarditis during the first two weeks [19]. Because of high nephrotoxicity risk for elderly, a narrow-spectrum monotherapy treatment with benzylpenicillin was preferred for these patients instead of combination of benzylpenicillin and gentamicin.

In conclusion, *S. dysgalactiae* subsp. *equisimilis* is a pathogen which very rarely causes cardiac device endocarditis. The two cases reported here emphasize the importance of considering endocarditis in elderly persons having cardiac devices together with the presence of unexplained bacteremia.

### Consent statement

Written informed consent was obtained from both of the patients for publication of this case report and accompanying images. A copy of the written consent is available for review by the Editor of this journal.

## Competing interest

The authors do not declare any conflicts of interest.

## Authors’ contributions

Dr Sari Rantala is a specialist in infectious disease and internal medicine in the Department of Internal medicine at Tampere University Hospital. She performed a literature review and drafted the manuscript and revised it. She treated both of the patients at Tampere University Hospital as infectious disease consultant. *S. dysgalactiae* subsp. *equisimilis* bacteremia was also a subject of her doctoral thesis. Dr Suvi Tuohinen is a cardiologist and treated patient two at Tampere University Hospital. She prepared the figures of transthoracic and transesophageal echocardiography and revised this manuscript. Both authors read and approved the final manuscript.

## Pre-publication history

The pre-publication history for this paper can be accessed here:

http://www.biomedcentral.com/1471-2334/14/174/prepub
